# INPP4B: the New Kid on the PI3K Block

**DOI:** 10.18632/oncotarget.260

**Published:** 2011-04-10

**Authors:** Irina U. Agoulnik, Myles C. Hodgson, Wayne A. Bowden, Michael M. Ittmann

**Affiliations:** ^1^ Herbert Wertheim College of Medicine, Miami, FL; ^2^ Baylor College of Medicine, Houston, TX

**Keywords:** cancer, PI3K, phosphatase, PTEN, tumor suppressor, oncoprotein, oncotarget

## Abstract

Dysregulation of phosphatidyl inositol signaling occurs in many cancers and other disorders. The lipid and protein phosphatase, PTEN (Phosphatase and Tensin homology protein on chromosome 10), is a known tumor suppressor whose function is frequently lost in various malignancies due to mutations in the coding region or genomic deletions. Recently, another lipid phosphatase, Inositol Polyphosphate 4-phosphatase type II (INPP4B), has emerged as a potential tumor suppressor in prostate, breast, and ovarian cancers and possibly in leukemia. We will review its structure and function, crosstalk with androgen receptor signaling, and regulation of INPP4B expression, as well as existing data about its role in cancer.

## INPP4B STRUCTURE AND FUNCTION

**Figure 1:**
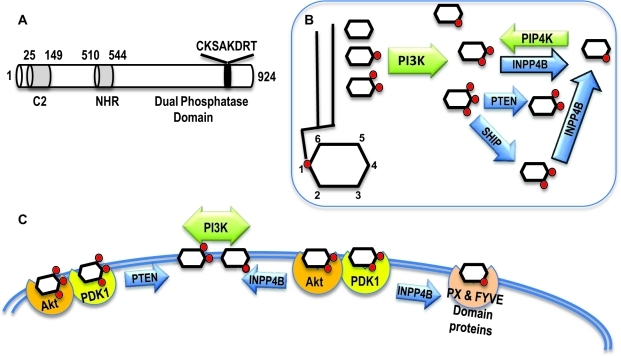
INPP4B structure and function A. Functional domains of INPP4B: C2 lipid binding domain amino acids 25-149, NHR domain amino acids 510-544, and putative Dual Phosphatase domain with the catalytic region C(X)_5_R. B. Network of kinases and phosphatases modifying inositol mono- and polyphosphates. Kinases are depicted in green and phosphatases in blue. C. The substrate and product of INPP4B enzymatic activity interact with various proteins changing their localization and activity.

INPP4B is one of many enzymes maintaining tight homeostasis of phosphoinositides in the cell. Phosphoinositides are produced by a large number of phosphatidylinositol kinases and dephosphorylated by lipid phosphatases with specific activities for phosphates at different positions of the inositol ring. INPP4B contains an N-terminal C2-lipid binding domain, internal NHR2 (Nervy Homology 2 domain), and a C-terminal phosphatase domain (Figure 1A). Human and mouse INPP4B C2 lipid binding domains exhibit over 91% identity. It has been shown that mouse INPP4B C2 domain preferentially binds phosphatidic acid and phosphatidylinositol 3,4,5-trisphosphate PI(3,4,5)P3 [1]. The NHR2 domain is a hydrophobic repeat that has been shown to mediate oligomerization and protein-protein interaction [2-4]. Although a role for this domain has yet to be determined it may mediate some interactions of INPP4B with other proteins. The C-terminal lipid phosphatase domain contains a CKSAKDRT (aa 842-849) motif conserved between type I and type II phosphatases that contains the catalytic active site (C(X)5R) of Mg^2+^ independent phosphatases such as protein tyrosine phosphatases, acid phosphatases, and notably dual specificity phosphatases capable of dephosphorylating both lipids and proteins. Mutation of the cysteine residue at position 842 to alanine in this motif renders INPP4B unable to dephosphorylate phosphatidylinositols [5].

The main substrate for INPP4B is phosphatidylinositol 3,4-bisphosphate (PI(3,4)P2) which it dephosphorylates on the D4 position generating phosphatidylinositol 3-phosphate (PI(3)P). Both the substrate and product of INPP4B lipid metabolism are important second messengers in the cell whose levels are controlled by a number of kinases and phosphatases.

## PI(3,4)P2 MEDIATED SIGNALING

PI(3,4)P2 is synthesized from the most abundant phosphatidylinositols in the cell, PI(3)P and PI(4)P, by Class I PI3K and PIP4K, respectively. In addition, the lipid phosphatase SHIP produces PI(3,4)P2 by dephosphorylating PI(3,4,5)P3 on the 5^th^ position (Figure 1B). The murine C2 domain of INPP4B preferentially binds to phosphatydic acid and PI(3,4,5)P3 *in vitro* [1]. However, despite the strong affinity for PI(3,4,5)P3, INPP4B fails to dephosphorylate this lipid. Gewinner *et al* reported that INPP4B is able to dephosphorylate PI(3,4)P2 *in vitro* and overexpression of INPP4B depleted PI(3,4)P2 in cells [5]. Similar to PI(3,4,5)P3, PI(3,4)P2 binds to the pleckstrin homology (PH) domains of both Akt and PDK1 and recruits them to the plasma membrane (Figure 1C). Depletion of PI(3,4)P2 in bone marrow derived mast cells, and stimulation of PI(3,4,5) production led to the recruitment of Akt to the cell membrane but failed to fully stimulate Akt activity [6]. Conversely, intracellular delivery of PI(3,4)P2 to SHIP−/− cells increased phosphorylation of S473, but not T308 of Akt [6]. In B-cells PI(3,4,5)P3 also contributes predominantly to T308 phosphorylation and membrane-associated activation of Akt, whereas PI(3,4)P2 contributes mostly to S473 phosphorylation and cytoplasmic activation of Akt [7]. Phosphorylation of Akt at T308 is associated with co-recruitment of Akt and PDK1 to PI(3,4,5)P3 at the plasma membrane and S473 phosphorylation has been attributed to the activity of mTORC2 [8]. In agreement with this data we observed that in prostate cancer cells depletion or overexpression of INPP4B regulated phosphorylation of S473 more strongly than T308 [9]. The PH domain of TAPP (tandem PH-domain containing protein) 1 and 2 proteins have also been shown to specifically bind PI(3,4)P2 [10]. TAPP1 is recruited to the plasma membrane following growth factor stimulation and has been implicated in actin cytoskeletal remodeling associated with cell migration [11]. Thus, INPP4B likely modulates cellular motility through the suppression of both Akt and TAPP1 activity. In addition, both TAPP1 and TAPP2 have been shown to be involved in the regulation of cellular insulin sensitivity [12]. Therefore these proteins may mediate and contribute to the intracellular responses to insulin that are coordinated by PI3K signaling and the synthesis of PI(3,4)P2 and PI(3,4,5)P3.

## PI(3)P MEDIATED SIGNALING

The INPP4B metabolite, PI(3)P, is significantly more abundant than PI(3,4) in quiescent cells. Synthesis of PI(3)P is predominantly regulated by class II and class III PI3K and is typically associated with endosomes, multivesicular bodies, and phagosomes [13]. Class III PI3K has been implicated as a potential tumor suppressor through its critical roles in autophagy and the prevention of genomic instability, endosomal sorting, lysosomal down regulation of mitogenic receptors, and regulation of cytokinesis during cell division [14-16]. Although the intracellular levels of PI(3)P are thought to remain relatively static, synthesis of PI(3)P at the plasma membrane has also been shown to be stimulated through extracellular signaling [13].

Similar to other plasma membrane associated phosphoinositides, PI(3)P is thought to act as a second messenger molecule. PI(3)P is recognized by FYVE (conserved in Fab1, YOTB, Vac1 and EEA1) and PHOX homology (PX) domains with high specificity (Figure 1C). FYVE and PX domain containing proteins are believed to mediate most of the downstream functions of PI(3)P. The recruitment of PI(3)P interacting proteins to specific cellular compartments is coordinated through both PI(3)P interaction and its associated proteins. It is possible that INPP4B locally regulates membrane PI(3)P content and the recruitment of effector proteins to specific cellular membranes. A Golgi specific isoform of INPP4B has been identified [1], but it remains to be determined what specific functions this isoform performs within the cell.

Although INPP4B has a clear role in regulating the activity of Akt in epithelial cells through the turnover of PI(3,4)P [5, 9, 17], what role it plays in modulating the activity of other PI3K signaling mediators remains to be determined. One especially interesting downstream mediator of PI3K signaling is serum- and glucocorticoid-regulated kinase 3 (SGK-3), which binds to PI(3)P via its PX domain [18]. Similar to Akt, PI3K activation of SGK3 is mediated through PDK1 [18, 19]. SGK3 has been implicated in PI3K dependent cancers in an Akt independent manner [19]. Expression of both SGK3 and SGK1 is induced by androgens in human prostate cancer cell lines and SGK3 activity may be stimulated by the INPP4B metabolite PI(3)P [18, 20]. Hence, SGK3 is an alternative signaling pathway that may compensate for inhibition of Akt signaling by INPP4B.

## REGULATION OF INPP4B EXPRESSION

**Figure 2:**
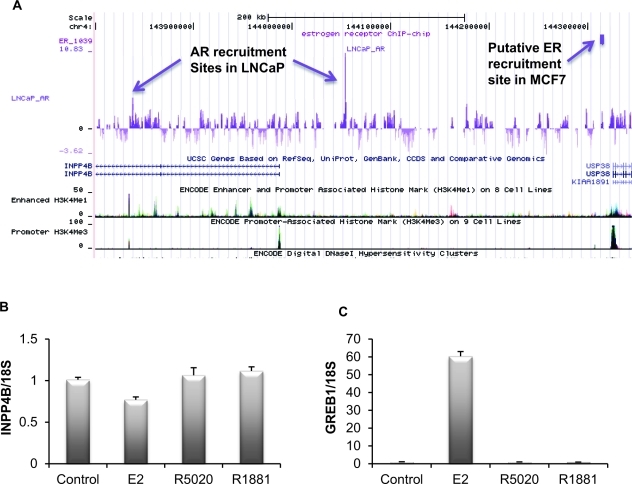
Regulation of the INPP4B expression A. Interrogation of the AR and ER recruitment sites in the vicinity of the INPP4B locus. Note AR recruitment in LNCaP cells upstream of the INPP4B promoter and in intron 2. Recruitment site for ERα in MCF7 cells is over 200 kb upstream and is immediately upstream of USP36 promoter as marked by the promoter methylation signature (H3K4Me). B. INPP4B expression is not hormonally induced in MCF-7 cells. MCF-7 cells grown in 10% charcoal stripped serum (CSS) for 48 hours were treated with ethanol (control), 10 nM estradiol (E2), 10 nM R5020, or 10 nM R1881. Cells were harvested 24 hours post-treatment, RNA was extracted, and *INPP4B* and *18S* expression was analyzed by quantitative RT-PCR. C. To control for estradiol gene regulation, *GREB1* expression was analyzed by quantitative RT-PCR (error bars denote ± S. E.) and normalized by *18S* expression.

At present, little is known on the specifics of INPP4B gene regulation. INPP4B is expressed in a wide array of tissue types unlike the related INPP4A, which is predominantly restricted to the brain. INPP4B is highly expressed in skeletal muscle, heart, brain, and pancreas, in addition to the epithelial cells of the breast and prostate glands [21]. To date, androgen driven regulation of INPP4B expression in human prostate cancer cell lines is the only known upstream regulator of INPP4B gene and protein expression [9]. A nonbiased screen for AR recruitment sites in LNCaP cells suggested two sites located in an upstream enhancer region and intron 2 (Figure 2A) that we have confirmed by ChIP assay [9, 22]. Interrogation of a dataset generated by Dr. Myles Brown's laboratory from a similar screen of ERα genomic recruitment in MCF-7 breast cancer cells showed no ERα recruitment to the INPP4B locus and corresponding lack of up- or downregulation of INPP4B expression (http://research4.dfci.harvard.edu/brownlab/datasets/index.php) (Figure 2A). Indeed, we observe that INPP4B expression is not regulated by estradiol, progestin, or androgens at the mRNA level in MCF-7 cells (Figure 2B). This is in agreement with Fedele *et al* who showed that INPP4B protein levels are not affected by estradiol in MCF-7 cells [17]. Although INPP4B does not appear to be hormonally regulated in breast cancer cells, its expression appears to be tightly associated with hormone receptor status. INPP4B LOH is frequently observed in hormone receptor negative breast cancers and loss of INPP4B protein is associated with loss of hormone receptors and basal-like breast cancers [5, 17]. Furthermore, INPP4B protein is only detected in ERα positive nonproliferative epithelial cells of the normal human breast [17]. Thus, in endocrine epithelial cancers, INPP4B may play a significant role in suppression and regulation of hormone receptor driven proliferation.

## CROSSTALK BETWEEN INPP4B AND AR SIGNALING PATHWAYS

Several reports have demonstrated a role for INPP4B in the regulation of Akt signaling. In addition, numerous studies have reported evidence of crosstalk between the AR and PI3K/Akt signaling pathways. INPP4B can thus exert its effect on AR signaling by inhibiting Akt and affecting its immediate phosphorylation targets. There is no clear consensus on the effect of Akt activity on AR function; it appears to be dependent on the cellular context and passage number and is promoter specific. Akt phosphorylates AR *in vitro* on S215 and S792 [ENST00000374690], and interacts directly with endogenous AR in LNCaP cells [23-25]. Activation of the PI3K/Akt signaling pathway has been shown to increase AR transcriptional activity in PSA-driven reporter assays and elevate PSA protein levels in LNCaP cells [25, 26]. Various downstream targets of Akt signaling can also interact with the AR to regulate its function in the prostate. β-catenin is part of the pro-proliferative Wnt signaling pathway, plays a vital role in cell-cell adhesion, and is positively regulated by Akt [27]. Activating mutations of β-catenin and/or inactivating mutations of its regulators are a common event in PCa as well as in other epithelial carcinomas [28-30]. It has been shown that β-catenin binds to AR, increasing its agonist dependent transcriptional activity [14, 15]. In turn, the AR has been shown to suppress β-catenin by limiting its interaction with transcriptional co-regulators such as TCF4 [31, 32]. In addition, pro-apoptotic Forkhead box transcription factors FOXO1a and FOXO3a are negatively regulated by Akt and affect AR signaling. FOXO1a down-regulation has been implicated in human prostate cancer. Ectopic expression of FOXO1a down-regulates endogenous levels of PSA and reduces androgen-mediated proliferation in LNCaP cells, suggesting that FOXO1a reduces AR transcriptional activity [33, 34]. In turn, AR negatively regulates FOXO1a transcription by interfering with its binding to DNA [35]. Thus, AR and FOXO1a seem to be mutually inhibitory. On the other hand, constitutive activation of another member of the forkhead box family, FOXO3a, can enhance AR transactivation and increase endogenous AR protein levels in LNCaP cells [36]. This complexity of AR regulation by Akt downstream targets may contribute to the differences observed in different cellular contexts.

**Figure 3:**
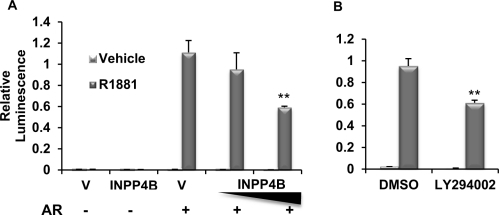
INPP4B reduces AR transcriptional activity A. PC-3 cells were transfected for 24 hours with vector control, 50 or 100 ng INPP4B, and the androgen-responsive GRE-luciferase reporter construct. Cells were then treated with ethanol or R1881, harvested, luciferase activity measured, and normalized for protein concentration. B. Cells were transfected as in A and treated with vehicle or R1881 with either DMSO or 20 nM LY294002. Activity was normalized to total protein. ** indicates statistically significant difference with p<0.01.

Suppressing PI3K/Akt signaling with the pan-PI3K inhibitor LY294002 or exogenous expression of PTEN, a negative regulator of Akt activity, can reduce AR signaling. PSA protein expression and secretion in PTEN negative LNCaP cells were decreased with overexpression of PTEN or treatment with LY294002 [37, 38]. In addition, transient expression of PTEN with wild-type AR suppresses AR activity on androgen-activated MMTV or GRE driven reporters in DU145 and PC-3 prostate cancer cell lines [37, 38]. Interestingly, the mechanism by which PTEN reduces AR activity may not be entirely via suppression of Akt signaling. Using coimmunoprecipitation, Lin *et al* showed direct interaction between PTEN and AR in LNCaP cells stably transfected with PTEN and that this interaction interferes with AR nuclear translocation independently of Akt [37]. However, another study found that PTEN-mediated suppression of AR function in PC-3 cells was dependent on protein expression of the Akt target FOXO1a [39]. These observations suggest that PTEN may regulate AR through both modulating Akt activity and direct interaction. Since AR regulates INPP4B expression and INPP4B in turn regulates Akt phosphorylation, we sought to determine if INPP4B modulates AR activity. We coexpressed INPP4B with AR and found that it reduces androgen dependent activation of GRE-luciferase reporter in PTEN-null PC-3 cells (Figure 3A) to a similar extent as inhibition of Akt signaling by LY294002 (Figure 3B). Using a mammalian two hybrid system we were unable to detect any interaction between AR and INPP4B (data not shown). This suggests that INPP4B exerts its effect at least in part through modulating the Akt pathway. Therefore both PTEN and INPP4B are able to modulate AR activity in PCa cells.

## INPP4B FUNCTION AS A TUMOR SUPPRESSOR

The first evidence of INPP4B involvement in tumorigenesis came from analysis of gene expression in the leukemic blasts from 132 patients to select for highly ranked class discriminators in pediatric acute lymphoblastic leukemia (*ALL*). Comparison of gene expression profiles in major subgroups revealed that INPP4B expression is increased 12.4 fold in *BCR-ABL* leukemia compared to all other *ALL* subgroups [40]. Two years later INPP4B was identified as a potential tumor suppressor in a non-biased screen for transcripts that inhibit transformation of human mammary epithelial cells (HMEC) [41]. Ninety percent of shRNAs that induced epithelial cell transformation were directed against 8 genes, including INPP4B [41]. This finding was corroborated in subsequent reports that described loss of INPP4B in breast and ovarian cancer, and that loss of INPP4B is associated with decreased patient survival [5]. Loss of INPP4B protein in breast cancer occurs most frequently in aggressive hormone receptor-negative basal-like breast carcinomas, with higher tumor grade, size, and increased proliferation. In HMECs and breast cancer cell lines INPP4B was able to suppress both basal [17] and IGF induced Akt phosphorylation [5], anchorage independent growth, invasion, and motility. Interestingly, depletion of both INPP4B and PTEN in HMECs resulted in cellular senescence, which could be alleviated by knockdown of p53 [5]. However, in mammary tumors INPP4B loss is observed more frequently in patients who have also lost PTEN [17].

Using immunohistochemistry we have shown highly significant downregulation of INPP4B protein in prostate cancers relative to benign prostate epithelium in radical prostatectomy specimens from men with clinically localized prostate cancer [9]. Of note, patients with decreased INPP4B levels in their prostate cancer tissues had significantly increased risk of biochemical recurrence. Consistent with these findings, a large scale analysis of DNA copy numbers, mRNA expression, and mutation analysis in 218 prostate tumors highlighted AR and PI3K as the most commonly altered pathways in primary and metastatic prostate cancers. For INPP4B, loss of expression or mutations were found in 8% and 47% of primary tumors and metastases respectively, while for PTEN similar changes were found in 4% of primary tumors and 42% of metastases suggesting a tumor suppressor role for INPP4B in prostate cancer [42]. We have shown that INPP4B is directly regulated by AR in LNCaP and VCaP prostate cancer cells. Somewhat differently from HMECs and breast cancer cells, INPP4B inhibits phosphorylation of Akt and its downstream target FOXO3a in prostate cancer cells with or without PTEN expression [9]. Furthermore, INPP4B depletion significantly increased proliferation of the PTEN negative prostate cancer cell line LNCaP. Thus, based on both clinical and biological evidence INPP4B appears to be a tumor suppressor gene in prostate cancer and is inactivated at rates similar to the classic tumor suppressor gene PTEN.

## CONCLUDING REMARKS

**Figure 4:**
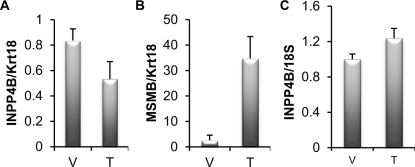
INPP4B expression is not induced by testosterone in mice A. Four month old male castrated mice were treated with vehicle (V) (n=5) or 1 μg testosterone (T) (n=9). Prostates were isolated 24 hours following treatment, RNA extracted and *Inpp4b* and *Krt18* expression was analyzed by quantitative RT-PCR. *Inpp4b* expression was correlated to *Krt18*, an epithelial specific marker and expression normalized to the castrated group. B. Brain tissue from the same mice were collected in parallel and analyzed for *Inpp4b* and *18S* expression. C. To control for testosterone gene regulation, *Msmb* expression was analyzed by quantitative RT-PCR (error bars denote ± S. E.).

Similar to various mouse models of cancer altering PTEN or Akt activity, valuable data will be obtained by modulating INPP4B expression in the mouse. However, there may be some substantial differences in INPP4B biology in the mouse compared to human. One of the immediate differences is lack of androgen regulation of INPP4B in mouse prostate. We tested if supplementation with androgen in castrated mice would upregulate *Inpp4b* expression in the prostate and found no significant increase (Figure 4A). Androgen signaling is well established in mouse prostate and we observed significant induction of the AR target gene *Msmb* in these animals confirming that testosterone treatment was successful (Figure 4B). Significantly, although the brain contains AR (tissue specific patterns of nuclear receptors; http://www.nursa.org), INPP4B expression in the mouse brain is also not affected by testosterone (Figure 4C).

## METHODS

### Mice and Tissue Isolation

Adult male FVB mice were obtained from Jackson laboratories (Bar Harbor, ME) and maintained in a temperature controlled room, with 12-h light, 12-h dark photocycle and fed Teklad global 18% protein rodent diet chow (Harlan, Indianapolis, IN) and fresh water *ad libitum*. Prostates were isolated 2 weeks after castration immediately following sacrifice using an Olympus SZ61 stereo microscope (Olympus, Center Valley, PA) and stored in RNA Later (Ambion, Austin, TX) at −80°C prior to RNA extraction.

### Castration and Testosterone Supplementation

To ablate endogenous testicular steroid hormones, four-month-old male FVB mice were castrated bilaterally. Castrated mice were given a single intrascapular subcutaneous injection of sesame oil (control group) or 1 μg testosterone in sesame oil two weeks following castration and sacrificed 24-h following injections.

### RNA Extraction and Real-Time PCR

Total RNA was extracted from isolated prostates using Trizol reagent (Invitrogen, Carlsbad, CA), as described by the manufacturer. First strand cDNA was synthesized using the SuperScript III First-Strand synthesis SuperMix for qRT-PCR (Invitrogen). The Roche Universal Probe library and primers were used to amplify the following mouse genes: *Inpp4b* (Forward: tgaccctgaggacattcagtt Reverse: attccaactgtggctcgttc, Probe 89), Cytokeratin 18 (*Krt18*) (Forward: agatgacaccaacatcacaagg Reverse: tccagaccttggacttcctc, Probe 78), and *Msmb* (Forward: cgtggtgttcatgtgacaaaa Reverse: ctcaaaggcctagtagcgttg, Probe 62). The following primer and probe sets were used for the human genes: *INPP4B* (Forward: gaaagcttccactcgtggtg Reverse: tgtttcgctggtttcaagg, Probe 63), *GREB1* (Forward: tgtggagtgcctgaagtgac, Reverse: ctcagcagagacgaagaaagg, Probe 73) and *18S* (Forward: gcaattattccccatgaacg, Reverse: gggacttaatcaacgcaagc, Probe 48). Real-time PCR analysis was carried out on a Roche 480 LightCycler.

### Transactivation of AR

PC-3 cells (ATCC, Manassas, Virginia) were seeded at 1x10^5^ cells / well in 24-well plates (BD Biosciences). Cells were transfected in triplicate in serum-free media using 1 ul per well Lipofectamine 2000 reagent (Invitrogen) with pCR3.1AR or pCR3.1, GRE-Luciferase, pCMV3XFLAG-INPP4B or pCMV3XFLAG. Four hours after transfection, media was replaced with one supplemented with charcoal-stripped serum and treated with either 10 nM R1881 or ethanol vehicle. Media was also supplemented with LY294002 or DMSO as indicated. The next day, firefly luciferase activity was assayed with the Dual-Luciferase Assay System (Promega) and normalized to total protein levels.
